# A Prospective Study of Marine Phytoplankton and Reported Illness Among Recreational Beachgoers in Puerto Rico, 2009

**DOI:** 10.1289/ehp.1409558

**Published:** 2015-09-18

**Authors:** Cynthia J. Lin, Timothy J. Wade, Elizabeth A. Sams, Alfred P. Dufour, Andrew D. Chapman, Elizabeth D. Hilborn

**Affiliations:** 1 Oak Ridge Institute for Science and Education (ORISE) Research Participation Program at the U.S. Environmental Protection Agency (EPA), Chapel Hill, North Carolina, USA; 2 Department of Epidemiology, UNC Gillings School of Global Public Health, Chapel Hill, North Carolina, USA; 3 Environmental Public Health Division, National Health and Environmental Effects Research Laboratory, Office of Research and Development, U.S. EPA, Research Triangle Park, North Carolina, USA; 4 Microbial Chemical Environmental Assessment Research Division, National Exposure Research Laboratory, Office of Research and Development, U.S. EPA, Cincinnati, Ohio, USA; 5 GreenWater Laboratories, Palatka, Florida, USA

## Abstract

**Background:**

Blooms of marine phytoplankton may adversely affect human health. The potential public health impact of low-level exposures is not well established, and few prospective cohort studies of recreational exposures to marine phytoplankton have been conducted.

**Objective:**

We evaluated the association between phytoplankton cell counts and subsequent illness among recreational beachgoers.

**Methods:**

We recruited beachgoers at Boquerón Beach, Puerto Rico, during the summer of 2009. We conducted interviews at three time points to assess baseline health, water activities, and subsequent illness. Daily water samples were quantitatively assayed for phytoplankton cell count. Logistic regression models, adjusted for age and sex, were used to assess the association between exposure to three categories of phytoplankton concentration and subsequent illness.

**Results:**

During 26 study days, 15,726 individuals successfully completed all three interviews. Daily total phytoplankton cell counts ranged from 346 to 2,012 cells/mL (median, 712 cells/mL). The category with the highest (≥ 75th percentile) total phytoplankton cell count was associated with eye irritation [adjusted odds ratio (OR) = 1.30; 95% confidence interval (CI): 1.01, 1.66], rash (OR = 1.27; 95% CI: 1.02, 1.57), and earache (OR = 1.25; 95% CI: 0.88, 1.77). In phytoplankton group-specific analyses, the category with the highest Cyanobacteria counts was associated with respiratory illness (OR = 1.37; 95% CI: 1.12, 1.67), rash (OR = 1.32; 95% CI: 1.05, 1.66), eye irritation (OR = 1.25; 95% CI: 0.97, 1.62), and earache (OR = 1.35; 95% CI: 0.95, 1.93).

**Conclusions:**

We found associations between recreational exposure to marine phytoplankton and reports of eye irritation, respiratory illness, and rash. We also found that associations varied by phytoplankton group, with Cyanobacteria having the strongest and most consistent associations.

**Citation:**

Lin CJ, Wade TJ, Sams EA, Dufour AP, Chapman AD, Hilborn ED. 2016. A prospective study of marine phytoplankton and reported illness among recreational beachgoers in Puerto Rico, 2009. Environ Health Perspect 124:477–483; http://dx.doi.org/10.1289/ehp.1409558

## Introduction

Harmful algal blooms occur when phytoplankton accumulate and negatively affect the environment and human or animal health. Harmful blooms are associated with a small subset of phytoplankton species. Out of 4,000 marine phytoplankton, it is estimated that some 200 are high biomass producers, and only ~80 are potential toxin-producers (Masó and Garcés 2006; Smayda 1997; Smayda and Reynolds 2003; Zingone and Enevoldsen 2000). Although naturally occurring in fresh, estuarine, and marine waters, the growth, toxicity, and geographic distribution of harmful algae have increased in part because of environmental factors such as nutrient enrichment and warmer water temperatures (Dyble et al. 2008; Moore et al. 2008; Paerl et al. 2001; Paerl and Huisman 2008, 2009; Sellner et al. 2003). Most epidemiologic studies of harmful algal blooms, specifically those generated by cyanobacteria, have been conducted at freshwater sites. In the United States, freshwater harmful algal blooms have been associated with waterborne disease outbreaks that include dermatologic, gastrointestinal, respiratory, febrile, ear, and eye symptoms (Dziuban et al. 2006; Hilborn et al. 2014; Yoder et al. 2004). The World Health Organization (WHO) has established guidelines for cell count categories associated with the risk of human health effects (Bartram and Chorus 1999). The lowest guidance level of 20,000 cyanobacterial cells per milliliter was derived from an epidemiologic study of freshwater cyanobacteria exposure (Pilotto et al. 1997). Currently, there is no federal regulation of cyanobacteria or cyanotoxin exposure for recreational waters in the United States; however, several state and local governments have established guidelines for exposure based on their own risk assessments or those of the WHO (Burch 2008).

Adverse human health outcomes have been associated with marine dinoflagellates, diatoms, and cyanobacteria (WHO 2003). For example, harmful algal blooms produced by *Karenia brevis*, a marine dinoflagellate, have been reported to produce brevetoxins that are associated with gastrointestinal and respiratory illnesses (Backer et al. 2003, 2005; Hlavsa et al. 2011; Kirkpatrick et al. 2010). *Lyngbya majuscula*, a benthic marine cyanobacterium, is known to produce toxins, such as debromoaplysiatoxin and lyngbyatoxin, and to cause acute dermal lesions among swimmers (Nagai et al. 1996; Osborne et al. 2001, 2007; Osborne and Shaw 2008; WHO 2003). The picoplanktonic *Synechococcus*, a cyanobacterium, has been reported to produce microcystins, a group of potent hepatotoxic cyanotoxins (Carmichael and Li 2006). A limited number of epidemiologic studies have investigated the effects of harmful marine algal exposures, and most of these have focused on blooms of *K. brevis* (Backer et al. 2003; Kirkpatrick et al. 2010). As a result, thresholds or concentrations of phytoplankton associated with adverse health effects are not well-established for marine waters.

Given the association between increasing ocean temperatures and increased frequency of harmful algal blooms around the world, there is a need to understand the impact of harmful algal blooms on human health as climate change progresses (Dale et al. 2006; Gingold et al. 2014; Moore et al. 2008; Peperzak 2005). The objective of the present study was to evaluate the association between phytoplankton cell counts and subsequent illness among recreational beachgoers at a tropical marine beach.

## Methods

We conducted a prospective study of beachgoers at Boquerón Beach, Puerto Rico, in the summer of 2009. We assessed the relationship between phytoplankton counts and the development of illness after recreational exposure. This study was in the context of the National Epidemiological and Environmental Assessment of Recreational (NEEAR) Water Study. A description of the study design, objectives, and protocols and a report of the associations between fecal indicator organisms and swimming-associated illness have been published (Wade et al. 2010b).

**Figure 1:**
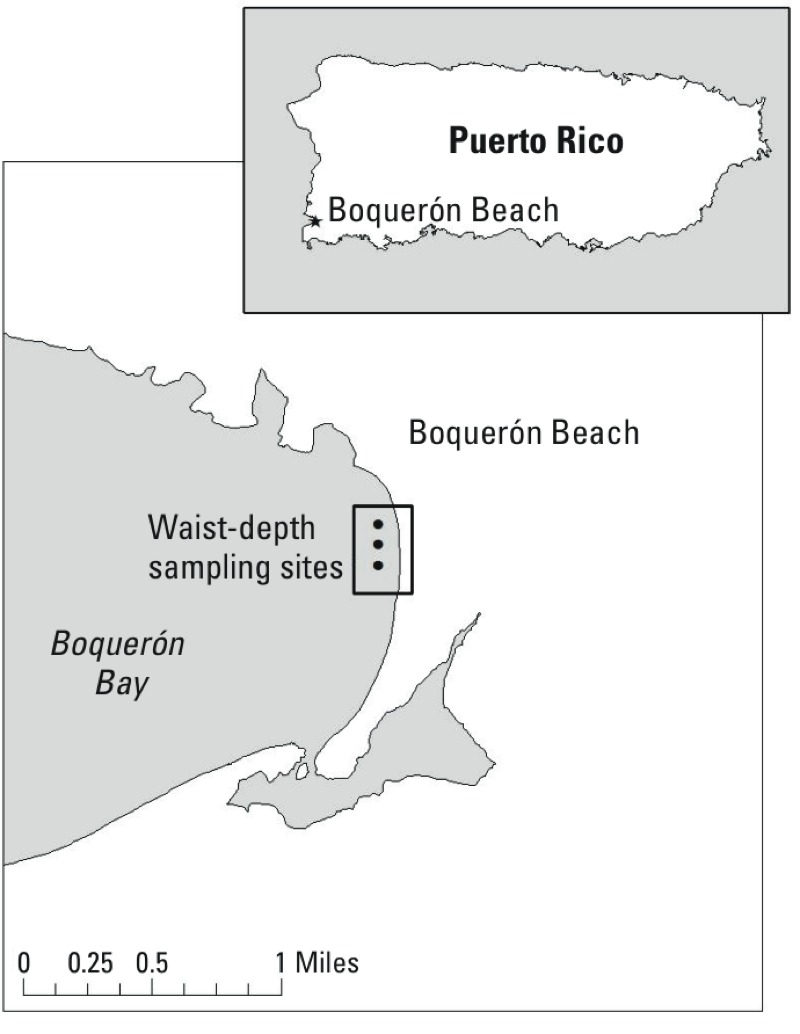
Water sampling sites at Boquerón Beach.

*Site description.* Boquerón Beach is located on the southwest coast of Puerto Rico (Figure 1). It is approximately 1 mile long and is located on Boquerón Bay, adjacent to the Caribbean Sea.

*Interviews.* We offered beachgoers enrollment in the study at the beach on summer weekends and holidays for a total of 26 days. Inclusion criteria consisted of *a*) an adult household member > 21 years of age; *b*) completion of three interviews (enrollment, beach exit, follow-up); and *c*) no previous participation in the study within the prior 28 days. An adult answered questions for all other household members at the beach. Interviews began at Boquerón Beach on 16 May 2009 and concluded on 2 August 2009.

Participants gave verbal informed consent for study participation in their chosen language, English or Spanish, and were interviewed by study personnel proficient in the language. All study materials were approved by the Institutional Review Board of the University of North Carolina at Chapel Hill. The enrollment interview collected information about demographics, baseline health, chronic health conditions, and contact information for follow-up. The exit interview collected information about water exposure and other activities in which the participants engaged while at the beach that day. Water exposure was ascertained with the following questions: “Did you immerse your body, not necessarily your head, in the water today?” “Did you put your face in the water or submerge your head in the water today?” and “Did you swallow the water?” The telephone interview, conducted 10–12 days later, recorded self-reported illness experienced since the beach visit. Participants were offered incentives, such as a cooler, a tote bag, or a beach-related item, to encourage completion of the exit interview. A US$25 check was provided to each household after the follow-up telephone interview was completed.

*Health end points.* The health end points were defined *a priori* and were similar to those previously studied in relation to recreational water quality and health (Colford et al. 2007; Prüss 1998; Wade et al. 2010a). They included incident cases of gastrointestinal (GI) illness, respiratory illness, rash (independent of sunburn), earache, and eye irritation occurring during the 10- to 12-day period between the beach visit and the follow-up telephone interview. The definitions for each illness are shown in Table 1. Signs and symptoms of each health end point were inquired about separately. For example, the following was asked for stomachache: “Have you or anyone else had a stomachache or abdominal cramping since the interview at Boquerón Beach?” Individual signs/symptoms of illness included the following: stomachache; diarrhea; nausea; vomiting; urinary tract infection; fever; headache; sore throat; cough; cold; runny or stuffy nose; earache, ear infection, or runny ears; watery eyes; eye infection; infected cut; and rash or itchy skin. For each question, participants could answer yes or no; they also had the option to refuse to answer or to say that they did not know.

**Table 1:** Definitions and exclusion criteria for outcomes that occurred during the 10- to 12-day period between the beach visit and the follow-up telephone interview.

Outcome	Definition	Baseline conditions^*a*^ excluded from analysis
Gastrointestinal illness	Any of the following: diarrhea (≥ 3 loose stools in a 24-hour period); vomiting; nausea and stomachache; nausea or stomachache, and interference with regular activities	Gastrointestinal illness or vomiting
Rash	Rash or itchy skin	Rash
Respiratory illness	Any 2 of the following: sore throat, cough, runny nose, cold, or fever	Sore throat
Earache	Earache, ear infection, or runny ears	Earache
Eye irritation	Eye irritation or infection, watery eye	Eye infection
^***a***^Baseline conditions occurred within 3 days before the beach visit.		

*Water samples.* Three fixed transects were selected at least 60 m apart to encompass the majority of the beach site. Water at sites along each transect was repeatedly sampled for marine phytoplankton counts and toxins; samples were collected from waist depth at each location at 1100 hours on each study day (Figure 1). All samples were refrigerated or placed on ice within 30 min of collection and were maintained under refrigeration during shipment to GreenWater Laboratories/CyanoLab (Palatka, Florida) for analysis. Water samples were combined into a daily composite sample and were quantitatively assayed for total and group phytoplankton cell counts (cells per milliliter) by an experienced phycologist who used the counting method described in *Standard Methods for the Examination of Water and Wastewater* (American Public Health Association 1998). The limit of detection using 5-mL samples at 100× magnification was 0.2 cells/mL. Water samples were also analyzed for two cyanobacterial toxins, lyngbyatoxin-a and debromoaplysiatoxin, using high-performance liquid chromatography–mass spectrometry (HPLC-MS) (Nagai et al. 1996; Osborne et al. 2008). These toxins were selected *a priori* based on the characteristics of the site; the limit of detection for both toxins was 1.0 ppb. Major phytoplankton groups were identified and included Cyanobacteria, Dinophyta (dinoflagellates), Bacillariophyta (diatoms), and miscellaneous other groups and morphotypes.

*Statistical analysis.* Multivariable logistic regression was used to evaluate the association between exposure to different categories of phytoplankton cell count and incidence of reported illness. The outcome was a binary indicator for each health end point as defined in Table 1. Each outcome was modeled separately. Each analysis excluded participants who reported having the outcome of interest in the 3 days before their beach visit.

Phytoplankton cell count was categorized as high (≥ 75th percentile), medium (> 25th to < 75th percentile), and low (≤ 25th percentile). The lowest category served as the referent in the regression models. Cell counts were considered in total and by phytoplankton group (e.g., Bacillariophyta, Cyanobacteria, Dinophyta). Picocyanophytes, a subgroup of Cyanobacteria, were examined separately from the Cyanobacteria group because they occurred at an order of magnitude higher than the other groups. To focus on those with recreational water contact, only participants who reported body immersion were included in models of the association between phytoplankton concentration and illness.

Covariates based on information from previous studies were considered for inclusion in the final model. Using frequency tables and chi-square tests, we identified factors associated with illness and/or water exposure to potential phytoplankton. These factors included age, sex, any other chronic illnesses, self-reported contact with algae, *Enterococcus* count, and digging in sand. After adjusting for different combinations of covariates, the association between phytoplankton exposure and incidence of reported illness varied by less than 0.1. The final model (adjusting for age and sex) was based on minimizing the Akaike information criterion (AIC) in order to balance model parsimony and fit. In sensitivity analyses, we considered other definitions of water exposure: head immersion and swallowing water. Duration of time spent in the water was evaluated as a potential effect measure modifier in stratified analyses.

## Results

*Respondent characteristics and demographics.* During the 26 study days, we included 15,726 individuals from 6,611 households. This represented 76% of the households initially approached and 96% of those completing the beach interview. There were slightly more female participants (55%), and nearly all (> 99%) participants self-identified as Hispanic. The average age was 30 years (range: < 1 to 92 years); children < 12 comprised < 20% of the study population. Approximately one quarter of all participants reported having a chronic illness: 13% reported having allergies, 11% reported having asthma, 5% reported having a chronic GI illness, and 3% reported having a chronic skin condition. Women reported more chronic illnesses, specifically chronic GI illness (6% women; 3% men; χ^2^ *p*-value < 0.0001) and allergies (14% women; 11% men; χ^2^ *p*-value = 0.0004). Age categories (0–4 years; 5–11 years; 12–19 years; 20–34 years; 35–64 years; ≥ 65 years) revealed differences in chronic illness (χ^2^ *p*-value = 0.0002); chronic GI illness increased with age, reports of allergies were infrequent among the youngest participants (0–4 years), and reports of asthma were most frequent among children < 12 years of age. Participants were excluded from analyses if they reported having the illness being evaluated in the 3 days before their beach visit. At enrollment, 8% of participants reported having a sore throat, and < 3% reported vomiting, other GI illness, rash, eye irritation, or earache in the previous 3 days. Table 2 summarizes the basic characteristics for all study participants and for those who reported body immersion in the water.

**Table 2:** Characteristics of study population by level of water contact.

Characteristic	*n* (%) of all participants (*n *= 15,726)	*n* (%) of participants reporting body immersion (*n *= 12,111)
Sex
Male	7,052 (44.8)	5,664 (46.8)
Female	8,654 (55.0)	6,431 (53.1)
Missing	20 (0.1)	16 (0.1)
Age category (years)
0–4	908 (5.8)	764 (6.3)
5–11	1,791 (11.4)	1,678 (13.9)
12–19	2,272 (14.5)	1,940 (16.0)
20–34	4,407 (28.0)	3,422 (28.3)
35–64	5,594 (35.6)	3,866 (31.9)
≥ 65	540 (3.4)	284 (2.3)
Missing	214 (1.4)	157 (1.3)
Ethnicity
Non-Hispanic	104 (0.7)	75 (0.6)
Hispanic	15,609 (99.3)	12,027 (99.3)
Missing	13 (0.1)	9 (0.1)
Visits to this beach each summer
0–1	4,592 (29.2)	3,507 (29.0)
2–5	7,533 (47.9)	5,841 (48.2)
> 5	3,600 (22.9)	2,762 (22.8)
Missing	1 (0)	1 (0)
Miles traveled to beach
0–20	5,161 (32.8)	3,878 (32.0)
20–60	5,597 (35.6)	4,339 (35.8)
60–100	2,795 (17.8)	2,162 (17.9)
> 100	1,106 (7.0)	863 (7.1)
Missing	1,067 (6.8)	869 (7.2)
Baseline health in the 3 days prior to beach visit
Vomiting	130 (0.8)	97 (0.8)
Other gastrointestinal illness	309 (2.0)	232 (1.9)
Sore throat	1,263 (8.0)	951 (7.9)
Rash	350 (2.2)	254 (2.1)
Eye irritation	153 (1.0)	98 (0.8)
Earache	224 (1.4)	164 (1.4)
Chronic illness
Any (gastrointestinal, skin, respiratory)	3,878 (24.7)	2,992 (24.7)
Chronic gastrointestinal illness	756 (4.8)	545 (4.5)
Chronic skin condition	548 (3.5)	401 (3.3)
Allergies	2,010 (12.8)	1,540 (12.7)
Asthma	1,684 (10.7)	1,352 (11.2)

*Beach visit activities.* Upon leaving the beach, 77% of all participants reported body immersion in the water, 64% reported head immersion, and 36% reported getting water in their mouths. Table 3 summarizes a sample of activities that participants reported engaging in at the beach. We analyzed the 12,111 participants (77%) who reported at least immersing their bodies in the water to improve the accuracy of exposure classification based on cell counts. As part of a sensitivity analysis, more substantial exposures were considered, including those who reported swallowing water as a marker of extreme exposure (*n* = 5,615). Among participants with body immersion, the mean duration spent in the water was just > 2 hr. One quarter of all participants spent ≥ 3 hr in the water, and the maximum time spent in the water was 8 hr.

**Table 3:** Activities reported at the beach exit interview.

Activity	*n *(%)
All	15,726 (100)
No water contact	2,995 (19.0)
Total time spent in water
< 60 min	5,547 (35.3)
60 to < 120 min	3,445 (21.9)
120 to < 180 min	2,835 (18.0)
≥ 180 min	3,892 (24.7)
Body immersion	12,111 (77.0)
Head immersion	10,074 (64.1)
Water in mouth	5,615 (35.7)
Played with algae/seaweed	2,499 (15.9)
Any contact with unknown animals	646 (4.1)
Dug in sand	3,699 (23.5)

*Illness after beach visit.* During the 10- to 12-day period between the beach visit and the follow-up telephone interview, respiratory illness was most commonly reported, with an overall incidence of 7%. The incidence was 5% for GI illness, 5% for rash, 3% for eye irritation, and 2% for earache.

*Water quality.* During the 26 study days, the median phytoplankton cell count was 712 cells/mL per day (range, 346–2,012 cells/mL). Of all groups identified, Bacillariophyta had the highest median (386 cells/mL per day). Cyanobacteria (excluding Picocyanophytes) had a median of 132 cells/mL per day. Picocyanophytes were only detected on 8 days but achieved a maximum count of 126,891 cells/mL. Dinophyta had a low median (37 cells/mL per day). Samples below the limit of detection (< 0.2 cells/mL) were assigned a value of 0 for calculating phytoplankton distributions. Table 4 summarizes the phytoplankton distribution over the study period.

**Table 4:** Phytoplankton distribution over 26 days.

Phytoplankton	Number (%) of days present	Minimum (cells/mL)	Maximum (cells/mL)	Mean (cells/mL)	Standard deviation (cells/mL)	25th percentile (cells/mL)	Median (cells/mL)	75th percentile (cells/mL)
All groups/phyla combined	26 (100)	345.7	2011.7	788.9	381.3	581.6	711.6	783.4
Bacillariophyta	26 (100)	128.6	619.4	377.5	102.6	301.2	385.9	442.5
Cyanobacteria (excluding Picocyanophytes)	24 (92)	0	1460.8	254.4	379.4	36.7	131.6	237.4
Picocyanophytes	8 (31)	0	126891.2	22606.4	38020.8	0	0	45126.8
Dinophyta	26 (100)	0.2	105.9	38.9	28.3	23.5	36.6	54.6
Samples below the limit of detection (< 0.2 cells/mL) were assigned a value of 0.								

Other phytoplankton groups were detected on < 8 study days at very low cell counts. These groups included Haptophyta (mean = 8.6 cells/mL), Chrysophyta (mean = 2.8 cells/mL), Rhodophyta (mean = 0.7 cells/mL), Euglenophyta (mean = 0.2 cells/mL), and Chlorophyta (mean = 0.1 cells/mL). See Appendix 1 for a list of the genera and morphotypes that were identified and their distribution according to phytoplankton group. Concentrations of lyngbyatoxin-a and debromoaplysiatoxin were below the limit of detection of 1.0 ppb in every sample.

*Enterococcus* colony forming units (CFU) and phytoplankton counts were not correlated (Spearman’s *r* = –0.14, *p*-value = 0.5). As previously reported by Wade et al. (2010b), low to moderate levels of fecal indicator bacteria were detected at Boquerón Beach, and the geometric means of the daily samples collected were all below the U.S. Environmental Protection Agency (EPA) guideline value of 35 CFU per 100 mL (Wade et al. 2010b). Spearman’s correlations between total phytoplankton counts and different environmental factors (e.g., wind speed and direction, air temperature, water temperature, turbidity) were all < 0.3 (data not shown).

**Figure 2:**
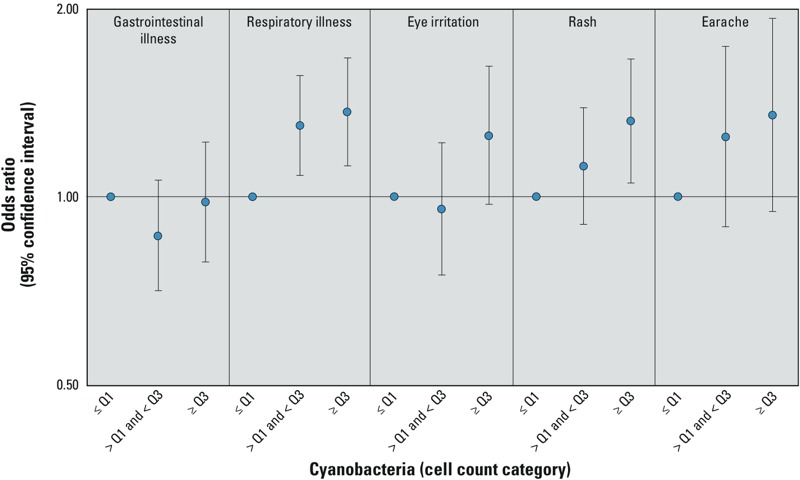
Associations between Cyanobacteria cell count and illness among beachgoers who reported body immersion in water. Models adjusted for age (as a continuous variable) and sex. Q1 = 25th percentile; Q3 = 75th percentile.

*Phytoplankton count and incident illness.* Among beachgoers who reported body immersion, the highest category of total phytoplankton cell count (≥ 75th percentile) was associated with eye irritation [adjusted odds ratio (OR) = 1.30; 95% confidence interval (CI): 1.01, 1.66], rash (OR = 1.27; 95% CI: 1.02, 1.57), and earache (OR = 1.25; 95% CI: 0.88, 1.77) (Table 5). Cyanobacteria cell counts were associated with respiratory illness, eye irritation, rash, and earache. These associations, although not all statistically significant at α = 0.05, strengthened with increasing Cyanobacteria cell count categories (Figure 2). In particular, respiratory illness, rash, and earache all had associations that increased relatively monotonically with each Cyanobacteria cell count category. Respiratory illness had the strongest association with all Cyanobacteria cell count categories. Table 5 shows the associations between phytoplankton group cell count and incident illness among beachgoers who reported body immersion.

**Table 5:** Associations between phytoplankton cell counts and incident illness occurring during the 10- to 12-day period between the beach visit and the follow-up telephone interview among beachgoers who reported body immersion in water.

Phytoplankton group	Gastrointestinal illness (*n *= 11,832)		Respiratory illness (*n *= 11,160)		Eye irritation (*n *= 12,013)		Rash (*n *= 11,857)		Earache (*n *= 11,947)	
	Cases (%)	OR (95% CI)	Cases (%)	OR (95% CI)	Cases (%)	OR (95% CI)	Cases (%)	OR (95% CI)	Cases (%)	OR (95% CI)
All groups combined
≤ Q1	174 (5.0)	1	227 (6.9)	1	114 (3.2)	1	156 (4.5)	1	57 (1.6)	1
> Q1 to < Q3	211 (4.5)	0.91 (0.74, 1.12)	315 (7.1)	1.03 (0.86, 1.23)	144 (3.0)	0.93 (0.72, 1.19)	199 (4.2)	0.95 (0.77, 1.18)	96 (2.0)	1.25 (0.90, 1.74)
≥ Q3	178 (4.9)	1.00 (0.80, 1.24)	244 (7.1)	1.02 (0.85, 1.24)	155 (4.2)	1.30 (1.01, 1.66)	206 (5.6)	1.27 (1.02, 1.57)	76 (2.1)	1.25 (0.88, 1.77)
Bacillariophyta
≤ Q1	142 (4.4)	1	189 (6.1)	1	110 (3.3)	1	170 (5.2)	1	54 (1.6)	1
> Q1 to < Q3	281 (5.0)	1.13 (0.92, 1.39)	405 (7.7)	1.28 (1.07, 1.54)	223 (3.9)	1.18 (0.94, 1.49)	279 (5.0)	0.97 (0.80, 1.18)	118 (2.1)	1.29 (0.93, 1.79)
≥ Q3	140 (4.7)	1.06 (0.83, 1.35)	192 (6.8)	1.10 (0.89, 1.35)	80 (2.7)	0.78 (0.58, 1.05)	112 (3.8)	0.73 (0.57, 0.94)	57 (1.9)	1.15 (0.79, 1.68)
Cyanobacteria
≤ Q1	173 (5.0)	1	187 (5.8)	1	114 (3.3)	1	144 (4.2)	1	55 (1.6)	1
> Q1 to < Q3	226 (4.4)	0.87 (0.71, 1.06)	354 (7.4)	1.30 (1.08, 1.56)	161 (3.1)	0.96 (0.75, 1.22)	236 (4.6)	1.12 (0.90, 1.39)	101 (2.0)	1.25 (0.89, 1.74)
≥ Q3	164 (5.0)	0.98 (0.79, 1.22)	245 (7.8)	1.37 (1.12, 1.67)	138 (4.1)	1.25 (0.97, 1.62)	181 (5.5)	1.32 (1.05, 1.66)	73 (2.2)	1.35 (0.95, 1.93)
Picocyanophytes
None	397 (4.8)	1	527 (6.7)	1	281 (3.3)	1	407 (4.9)	1	155 (1.8)	1
Any	166 (4.7)	0.96 (0.80, 1.16)	259 (7.8)	1.13 (0.97, 1.32)	132 (3.7)	1.10 (0.89, 1.36)	154 (4.3)	0.90 (0.74, 1.09)	74 (2.1)	1.11 (0.83, 1.47)
Dinophyta
≤ Q1	128 (4.5)	1	192 (7.2)	1	96 (3.3)	1	151 (5.3)	1	64 (2.2)	1
> Q1 to < Q3	275 (5.3)	1.22 (0.98, 1.51)	358 (7.2)	1.01 (0.84, 1.21)	185 (3.5)	1.04 (0.81, 1.33)	243 (4.6)	0.84 (0.68, 1.04)	87 (1.6)	0.72 (0.52, 1.01)
≥ Q3	160 (4.3)	0.97 (0.76, 1.23)	236 (6.7)	0.95 (0.78, 1.15)	132 (3.5)	1.05 (0.80, 1.37)	167 (4.4)	0.82 (0.65, 1.03)	78 (2.1)	0.93 (0.67, 1.30)
Abbreviations: CI, confidence interval; OR, adjusted odds ratio; Q1, 25th percentile; Q3, 75th percentile. Models adjusted for age (as a continuous variable) and sex. Denominator for the case percentage is the total number in the exposure group.										

Picocyanophytes were not associated with any subsequent illness among participants who reported body immersion in the water (Table 5). However, among those who reported swallowing water (*n* = 5,615), the presence of Picocyanophytes was significantly associated with earache (OR = 1.62; 95% CI: 1.14, 2.30), which was reported by 3.3% (*n* = 56) of those exposed and 2.1% (*n* = 83) of those unexposed. Similarly, among participants who reported swallowing water, the highest category of Cyanobacteria cell count was associated with earache (OR = 1.75; 95% CI: 1.09, 2.82), which was reported by 3.2% (*n* = 52) of those exposed to the highest category and 1.8% (*n* = 29) of those exposed to the lowest category.

As shown in Table 5, the medium category of Bacillariophyta was associated with respiratory illness (OR = 1.28; 95% CI 1.07, 1.54); however, the odds ratio did not increase for the highest category (OR = 1.10; 95% CI: 0.89, 1.35). Exposure to Dinophyta was not associated with any illness.

To assess potential effect measure modification by the length of time spent in water, we conducted analyses stratified by number of hours spent in the water (< 1 hr, 1 to < 2 hr, 2 to < 3 hr, ≥ 3 hr). We also restricted our analysis to other categories of water exposure (head immersion, swallowing water). The results were similar in the stratified analyses, with the exception of a stronger earache association with Cyanobacteria and Picocyanophytes after restricting to participants who reported swallowing water (data not shown).

## Discussion

We report the results of a prospective evaluation of the health effects associated with recreational water exposure to marine phytoplankton in the absence of a harmful algal bloom. Given the popularity of visiting beaches (Leeworthy et al. 2005) and the apparent increase in harmful algal blooms around the world (Sellner et al. 2003), we sought to better understand the effects of marine phytoplankton on human health. We found an association between total phytoplankton cell count and incident illness: specifically, eye irritation and rash. These outcomes have also been associated with freshwater blooms (Billings 1981; Pilotto et al. 1997; Rapala et al. 2005; Walker et al. 2008).

Our study design established a temporal sequence between exposure and outcome. By having interviews performed at different time points, participants did not have to wait a long time to recall their experiences, and their exposure response could not be influenced by any subsequent illness. The high participation rate (> 75%) reduced the possibility of selection bias. After considering a range of potential confounders, we only adjusted for age (as a continuous variable) and sex in order to balance model parsimony and fit. Because our final model produced similar results to those of the full model (adjusting for age, sex, any other chronic illnesses, self-reported contact with algae, *Enterococcus* count, and digging in sand), there did not appear to be a major bias due to confounding (data not shown).

Our study design allowed one adult to answer questions for all other household members at the beach. Although there was a possibility for responder bias or misinformation, there was an average of only 3.2 individuals per household, and other household members often assisted with the questionnaire responses. A limitation of our self-reported outcome data was a lack of specific details about some of the illnesses. Therefore, it was difficult to confirm the etiology of the illness on the basis of the participant responses alone. For example, we could not necessarily distinguish among rashes as being associated with cnidarians, sea lice, cercariae, salt water itself, or even something completely unrelated to the beach visit.

Cyanobacteria concentration was associated with all illnesses except GI illness; the odds of illness increased with cell count category. Our findings were consistent with reports of skin and eye irritations associated with *Lyngbya majuscula* blooms (Osborne et al. 2007; Osborne and Shaw 2008). Despite these similar illnesses, the *Lyngbya*-associated toxins, debromoaplysiatoxin and lyngbyatoxin-a, were below the limit of detection in all samples, and *Lyngbya* comprised only 3% of total planktonic Cyanobacteria among samples (see Appendix 1). Debromoaplysiatoxin and lyngbyatoxin-a are photolabile and are unlikely to persist in the water column (Moikeha et al. 1971). It is possible that people had contact with toxins or toxic material in the water or on the ocean floor because we did not sample the seabed or measure other cyanotoxins potentially associated with Cyanobacteria.

Unlike previous epidemiologic studies of freshwater cyanobacterial blooms (Lévesque et al. 2014; Pilotto et al. 1997), GI illness was the only illness that did not appear to be associated with marine Cyanobacteria in the absence of blooms, even when we restricted the analysis to participants who reported swallowing water. Although the lack of association with GI illness may have been because of low Cyanobacteria cell counts, health effects may also differ after exposure to communities of Cyanobacteria in fresh and marine waters. The maximum Cyanobacteria cell count (excluding Picocyanophytes) was 1460.8 cells/mL. In epidemiologic studies conducted at freshwater sites, illnesses were associated with cyanobacterial counts > 5,000 cells/mL (Pilotto et al. 1997) and at counts < 20,000 cells/mL relative to no water contact (Lévesque et al. 2014). More work is needed to define Cyanobacteria concentrations that are safe for human health in marine waters.

We analyzed Picocyanophytes separately because of the different orders of magnitude of the cell counts. Although most of the cyanobacteria literature describes marine picoplankton as nontoxic, there are some reports of toxic effects caused by homogenized *Synechococcus* and *Synechocystis* and their extracts (Martins et al. 2005, 2007; Walsh et al. 2008). Microcystins have been shown to cause adverse health effects (Codd et al. 1999; Falconer 1999; Giannuzzi et al. 2011), and a study of *Synechococcus* strains suggested that some marine picoplankton may be capable of synthesizing microcystins (Carmichael and Li 2006).

We observed an association between earache and Cyanobacteria among those who reported swallowing water. This finding is consistent with the positive but nonsignificant association we estimated among all those who immersed themselves in water. In the context of earache, we hypothesize that the stronger association, when restricted to those who swallowed water, may reflect more frequent head immersion and more intense exposure overall rather than being a direct consequence of swallowing water. Earache has been associated with swimming, especially when the head is immersed (Wade et al. 2013). The presence of any Picocyanophytes (vs. none detected) was associated with earache among participants who reported swallowing water. To our knowledge, this association has not been previously reported.

Associations with specific outcomes varied among other phytoplankton groups. Bacillariophyta cell counts in the 25th to 75th percentile range, but not counts above the 75th percentile, were significantly associated with respiratory illness when compared with counts below the 25th percentile. To our knowledge, Bacillariophyta have not been associated with respiratory illness in previous studies. Previous studies of marine diatoms, such as those of the genus *Pseudo-nitzschia* that produce domoic acid, have focused on adverse outcomes occurring after ingestion of contaminated shellfish rather than on recreational water exposure (Van Dolah 2000).

Because phytoplankton cell counts were low, we cannot be confident that associations with health outcomes were a result of phytoplankton exposure alone, or if phytoplankton were markers for other unmeasured causative factors, such as potentially pathogenic microbes or physical-chemical conditions associated with marine phytoplankton. In addition, we are unable to rule out noncausal mechanisms related to chance or bias (e.g., uncontrolled confounding, selection bias, information bias). Although phytoplankton may provide nutrients and substrates for the survival of microbial communities, there is limited knowledge on the occurrence of phytoplankton-associated pathogens (Brettar et al. 2007; Maugeri et al. 2004).

We categorized cell count *a priori* as high (≥ 75th percentile), medium (> 25th to < 75th percentile), and low (≤ 25th percentile) because health guidelines for concentrations of marine phytoplankton have yet to be established. As a result, it is possible that the highest cell count category was actually below any level that could cause potential adverse health effects. For example, although we did not find an association of illness with Dinophyta, maximum cell counts for Dinophyta were only 105.9 cells/mL. In contrast, epidemiologic studies of the marine dinoflagellate, *K. brevis*, and associated respiratory illness measured maximum cell counts ranging from 8,120 cells/mL (Backer et al. 2003) to 121,000 cells/mL (Backer et al. 2005).

We reported cell counts per milliliter of water so that our findings could be compared with those of previous studies and the WHO guidelines for freshwater exposures (Bartram and Chorus 1999). A limitation of our phytoplankton assessment was that we had no information on cell size to calculate biomass concentrations. Cell size can influence total phytoplankton exposure. For example, a few large cells of one species may contribute more to the overall biomass than many small cells of a different species (Hillebrand et al. 1999).

Finally, our study participants spent a large amount of time in the water; half of them spent ≥ 2 hr in the water. It is possible that we observed associations between health outcomes and low phytoplankton counts, in the absence of active phytoplankton blooms, because the participants spent so much time in the water. However, the associations did not vary significantly when stratified by total time in the water (data not shown).

Our results offer insight into the potential health effects of marine phytoplankton in the absence of a harmful algal bloom. Although some associations could be due to chance or bias, most seem plausible based on the existing literature. The evaluation of health effects associated with recreational exposure to marine phytoplankton at sub-bloom concentrations warrants further investigation.

## Conclusions

We found associations between recreational exposure to marine phytoplankton and subsequent reports of eye irritation, respiratory illness, earache, and rash at a tropical beach in the absence of an algal bloom. In addition, we found that associations varied by phytoplankton group, with Cyanobacteria having the strongest associations with most of the outcomes assessed.

### Appendix 1: Genera/morphotypes by phytoplankton group (percentage within each group)

**Bacillariophyta (69% of total phytoplankton)**

Pennate diatom (37); Nitzschia (12); Navicula (7); Licmophora (5); Bacillaria (4); Cylindrotheca/Nitzschia (4); Amphiprora (4); Psammodictyon (4); Chaetoceros (3); Centric diatom (3); Diploneis (2); Amphora (2); Rhizosolenia (2); Amphiprora/Plagiotropis (1); Actinocyclus/Coscinodiscus (1); Proboscia (1); Skeletonema (1); Cocconeis (1); Gyrosigma/Pleurosigma (1); Actinoptychus (1); Bacteriastrum (1); Diatom (1); Paralia (1); Achnanthes (< 1); Plagiotropis (< 1); Pseudo-nitzschia (< 1); Triceratium (< 1); Attheya (< 1); Guinardia (< 1); Gyrosigma (< 1); Odontella (< 1); Peralia (< 1); Tropidoneis (< 1)

**Dinophyta (13% of total phytoplankton)**

Dinoflagellate (67); Gonyaulax (16); Ceratium (7); Prorocentrum (7); Amphidinium (1); Dinophysis (1); Protoperidinium (1)

**Cyanobacteria (9% of total phytoplankton)**

Cyanophyte filament (32); Pseudanabaena (26); Picocyanophyte (10); Synechococcus (5); Synechocystis (5); Cyanophyte cell pair (5); Phormidium (4); Lyngbya (3); Trichodesmium (3); Aphanothece (1); Johannesbaptistia (1); Komvophoron (1); Cyanophyte colony (1); Cyanophyte unicell, sphere 2.5–5 μm (1)

**Miscellaneous (8% of total phytoplankton)**

Unknown flagellate (29); Unicell, sphere 2.5–5 μm (26); Unicell, oval 2.5–5 μm (14); Unknown unicell (11); Unicell, oval 5–7.5 μm (10); Microflagellate (4); Unicell, oval/rod 2.5–5 μm (3); Unicell, sphere 5–7.5 μm (1); Unknown filament (1)

**Haptophyta (0.8% of total phytoplankton)**

Haptophyte flagellate (100)

**Euglenophyta (0.6% of total phytoplankton)**

Euglenophyte (80)

Euglena/Eutreptiella (20)

**Chrysophyta (0.2% of total phytoplankton)**

Chrysophyte flagellate (100)

**Rhodophyta (0.2% of total phytoplankton)**

Rhodophyte filament (100)

**Chlorophyta (0.1% of total phytoplankton)**

Chlorophyte filament (100)
